# A short-term, high fat diet impairs cardiac high energy phosphate metabolism, without change in cardiac function

**DOI:** 10.1186/1532-429X-11-S1-O72

**Published:** 2009-01-28

**Authors:** Cameorn J Holloway, Yaso Emmanuel, Lowri E Cochlinl, Cezary Szmigielski, Lindsay M Edwards, Jane M Francis, Stefan Neubauer, Kieran Clarke

**Affiliations:** University of Oxford, Oxford, UK

**Keywords:** Left Ventricular Function, Diastolic Function, Heart Failure Patient, Calorie Intake, Phosphate Metabolism

## Introduction

Heart failure patients have low cardiac phosphocreatine/ATP (PCr/ATP) ratios, which may be related to elevated circulating free fatty acids (FFAs).

## Purpose

We tested whether raising plasma FFAs, using diet, causes abnormalities in cardiac energetics or function.

## Methods

Healthy males (n = 16, age 22 ± 1 years), recruited from the University of Oxford, were randomised to five days of a high fat diet (HFD) containing 75 ± 1% of calorie intake through fat consumption, or an isocaloric control diet, providing 23 ± 1% of calorie intake as fat. In a cross-over design, subjects undertook the alternate diet after a two week wash out period. Cardiac 31P magnetic resonance (MR) spectroscopy was performed to assess PCr/ATP before and after the diets. MR imaging and echocardiography were performed to assess left ventricular function.

## Results

**Figure 1:**
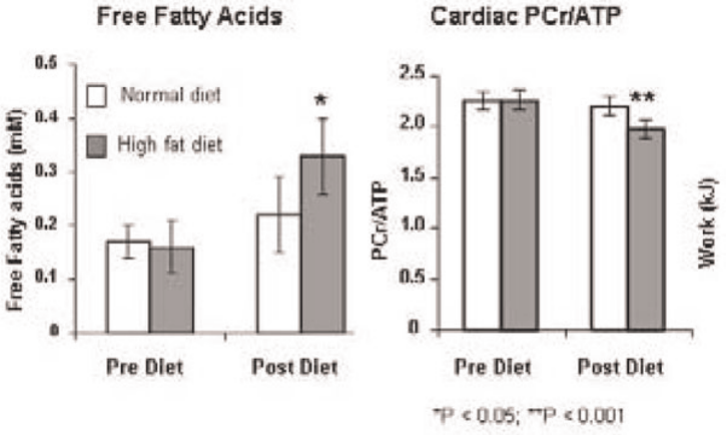
Figure 1

Subjects on the HFD had a two-fold elevation in plasma FFAs, 12% lower cardiac PCr/ATP with no change in cardiac systolic or diastolic function. Figure 1.

## Conclusion

We have shown a short term, high fat diet raised plasma FFA concentrations, impaired myocardial energetics without effect on systolic or diastolic function. This suggests that high plasma FFAs may be detrimental for heart in normal subjects and shows a potential mechanism of impairment in heart failure patients.

